# StrayCare Metro: Evaluation of a Targeted Cat Desexing Program to Manage Free-Roaming Cats

**DOI:** 10.3390/ani16081216

**Published:** 2026-04-16

**Authors:** Gemma C. Ma, Sarah Zito, Brooke P. A. Kennedy

**Affiliations:** 1 School of Veterinary Science, The University of Sydney, Camperdown, NSW 2050, Australia; 2 Royal Society for the Prevention of Cruelty to Animals New South Wales, Yagoona, NSW 2199, Australia; 3 RSPCA Australia, Deakin, ACT 2600, Australia; szito@rspca.org.au; 4 School of Environmental and Rural Science, University of New England, Armidale, NSW 2350, Australia; bkenne27@une.edu.au

**Keywords:** *Felis catus*, domestic cat, free-roaming, targeted desexing, surgical sterilization, population management, neutering, non-lethal control

## Abstract

Background: Free-roaming cats in Australian cities contribute to wildlife impacts, community concerns, and high shelter intake. We used an observational pre–post evaluation study design of a targeted cat desexing program (“StrayCare Metro”) delivered with councils and community partners in four local government areas (LGAs) of Greater Sydney (2022–2024). Methods: Program records documented cat enrolments and services; council and state databases supplied annual shelter intake, euthanasia, and cat-related complaints; and transect drives in two LGAs (2021 and 2024) estimated cat encounter rates and population density. The analysis did not include control LGAs. Results: The program desexed 1225 cats; among enrolled cats not already microchipped, 72% received a microchip and 28% declined despite this being offered for free. Compared with pre-program baselines, annual council shelter intake decreased by 49–73% within LGAs (61% overall), with concurrent reductions in euthanasia. Cat-related complaints declined in three LGAs (47–64%) but increased in one. Transect drives indicated substantial declines in cat encounter rates in Blue Mountains (51%) and Campbelltown (35%) and lower density estimates in both surveyed LGAs. Conclusions: A collaborative targeted desexing approach was associated with large reductions in council pound intake, euthanasia, and, in most areas, nuisance complaints, alongside independent indications of reduced free-roaming cat density.

## Simple Summary

Unmanaged free-roaming urban cat populations can threaten wildlife, cause concern in communities, and overwhelm shelters. We evaluated a free cat desexing (surgical sterilization) program—StrayCare Metro—run by an animal welfare charity across four areas of Greater Sydney, Australia, from 2022 to 2024. The StrayCare Metro program focused on engaging with people caring for unowned cats and households with many entire cats and included help with transport and microchipping. In total, 1225 cats were desexed. Shelter intake and euthanasia fell after the program began, and cat-related complaints to the council decreased in three areas. Standardized street counts in two areas saw fewer cats on the same routes in 2024 than in 2021, suggesting lower population density. Together, these findings indicate that targeted, well-supported cat desexing programs, delivered collaboratively with councils and community groups, can improve outcomes for cats and their caregivers, the community, and the environment.

## 1. Introduction

Australia is home to many domestic cats living in and around human communities; some of these are companion cats who are directly dependent on a specific person who considers themselves the owner (owned cats), some are cared for by people who do not consider themselves the owner of the cat (semi-owned cats), and some live around human communities and have some indirect dependence and casual and temporary interactions with people (unowned cats) [1]. Feral cats are unowned, unsocialized, have no relationship with or dependence on humans, and reproduce in the wild [1]. Feral cats are not discussed in this paper. Free-roaming cats who have no identifiable owner are often referred to as ‘stray’ cats in Australia, especially by those who provide them with care and those for whom they create a nuisance. However, the term ‘stray’ is problematic, as these cats may be semi-owned, unowned, or indeed owned cats who are lost or allowed to roam. In this paper, we refer to domestic cats who have no identifiable owner but are indirectly or directly dependent on people as ‘unowned cats’. Semi-owned cats are considered to be a subset of unowned cats; these cats have a person or people caring for them, but do not have a person who considers themselves the owner.

There are an estimated 5.8 million owned companion cats in Australia [2]. The number of unowned cats is unknown, with estimates ranging from 0.7 to 2 million (based on the calculation commonly used to estimate stray cat numbers in a community of 60–100 cats per 1000 human residents) [3,4,5,6,7].

Unowned cat populations and numbers vary across the huge variety of different environments in Australia [8]. Cat population density is influenced by many factors, including environment, climate, the socio-economic context of the area, and human population densities [6,9,10]. Where unowned cats are provided with support by humans (including shelter, medical care, and supplementary food, either directly via intentional feeding or indirectly through scavenging), there are fewer constraints on their reproduction and survival, leading to very dense populations and rapid population growth in some instances [8,10,11,12].

Free-roaming cat populations in Australia are associated with many concerns, including the threat to Australia’s native wildlife [13,14,15,16], cat welfare [1,12,14,17,18], involvement in the spread of disease (to other animals and people) [11,19,20], and behaviors that are undesirable to the human community (e.g., fighting, soiling, and noise) [17,21,22].

Tens of thousands of cats are admitted to animal shelters (including council pounds) across Australia each year—far exceeding the number that can be successfully rehomed [3,4,23,24]. This situation gives rise to potential issues such as compromised cat welfare during confinement in shelter environments [25,26,27], high rates of euthanasia, and resulting ethical conflicts. These challenges can also lead to substantial moral distress and psychological strain amongst shelter personnel and others involved in cat management, impacting their well-being and professional longevity [22,28,29,30]. In addition to these social costs, cat management is associated with substantial financial and human resourcing costs for animal welfare organizations, local government, and the veterinary profession [1,22,31,32].

Managing urban free-roaming cat populations is an issue that poses significant challenges; there are complex socio-environmental factors that need to be considered [21,33,34], and cat population density may be high [9,10]. Cat semi-owners and cat owners overwhelmed with multiple entire cats can inadvertently support the persistence of and even increase unowned cat populations. The practice of cat semi-ownership is reported to be relatively common in Australia [35,36,37]. Semi-owned cats are reported to make up the majority of cats entering animal welfare shelters and council pounds [3,23].

Addressing cat semi-ownership through targeted desexing programs and education could be a crucial part of managing cat populations and reducing shelter admissions [34,35]. Semi-owners are more likely to live in urban and lower socio-economic areas, as well as already own cats [34]. They often face barriers to sterilization or claiming ownership, including cost and trust issues, and are often caring for multiple cats, which exacerbates the costs of accessing services [34,35,38]. Entire owned cats and owned cats who are desexed after they have already produced kittens also contribute to cat overpopulation and the persistence of unowned cat populations [39,40,41]. Hence, cat management approaches that are holistic and address the contribution of all domestic cat sub-populations (owned, semi-owned, and unowned) might be more effective at reducing overall free-roaming cat population density. Studies have demonstrated that cat population management strategies focusing on desexing semi-owned and unowned cats, in addition to entire owned cats, can have positive outcomes such as reducing cat intake to animal shelters, rescue or welfare organizations, and pounds and can decrease cat nuisance in the community [7,22,42,43]. Recent mathematical modeling of cat populations in the UK indicated that sterilizing owned cats not only influences the population dynamics of owned cats but also has significant effects on feral, stray, and shelter cat sub-populations [41], highlighting the importance of considering the interlinked nature of cat sub-populations.

This study aimed to evaluate a targeted cat desexing program—StrayCare Metro—implemented in four local government areas within the Greater Sydney region of New South Wales, Australia, to inform the design and implementation of evidence-based cat management interventions.

## 2. Materials and Methods

### 2.1. Study Sites

**Figure 1:**
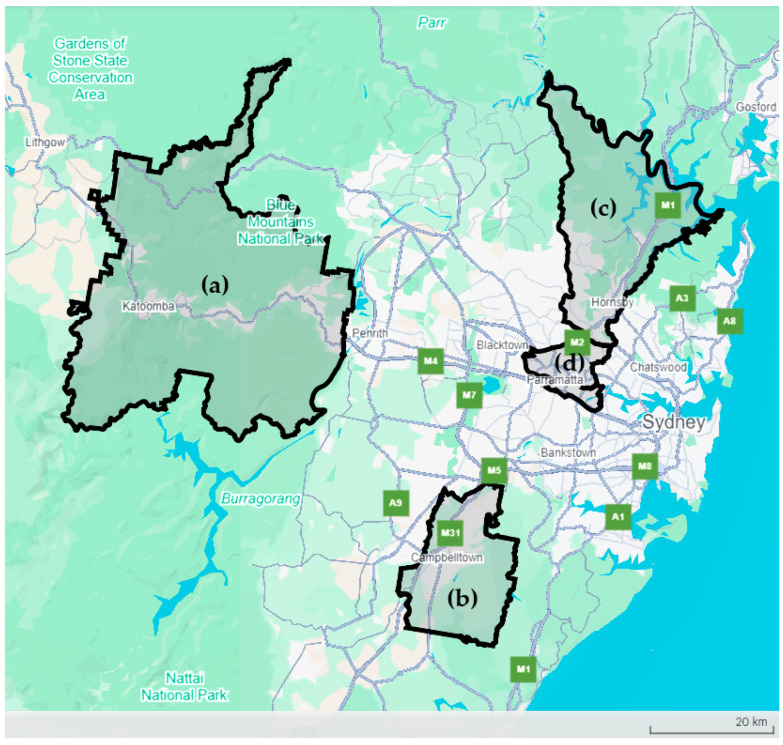
Map of the Greater Sydney region of New South Wales, Australia, showing the four local government areas where the StrayCare Metro targeted cat desexing program was implemented: (a) Blue Mountains, (b) Campbelltown, (c) Hornsby, and (d) Parramatta (Google).

The StrayCare Metro program was conducted in four local government areas (LGAs) within the Greater Sydney region of New South Wales (NSW), Australia, by the Royal Society for the Prevention of Cruelty to Animals NSW (RSPCA NSW) between 2022 and 2024. Sydney is the capital city of the state of NSW on Australia’s east coast. The Greater Sydney region covers around 1.3 million hectares with a human population of approximately 5.2 million [44]. Sydney has a temperate climate with mean daily temperatures between 11 and 23 °C and annual rainfall of around 1200 mm [45]. The program was conducted in the LGAs of Blue Mountains, Campbelltown, Hornsby, and Parramatta (Figure 1). Control LGAs were not included.

### 2.2. The StrayCare Metro Program

**Figure 2:**
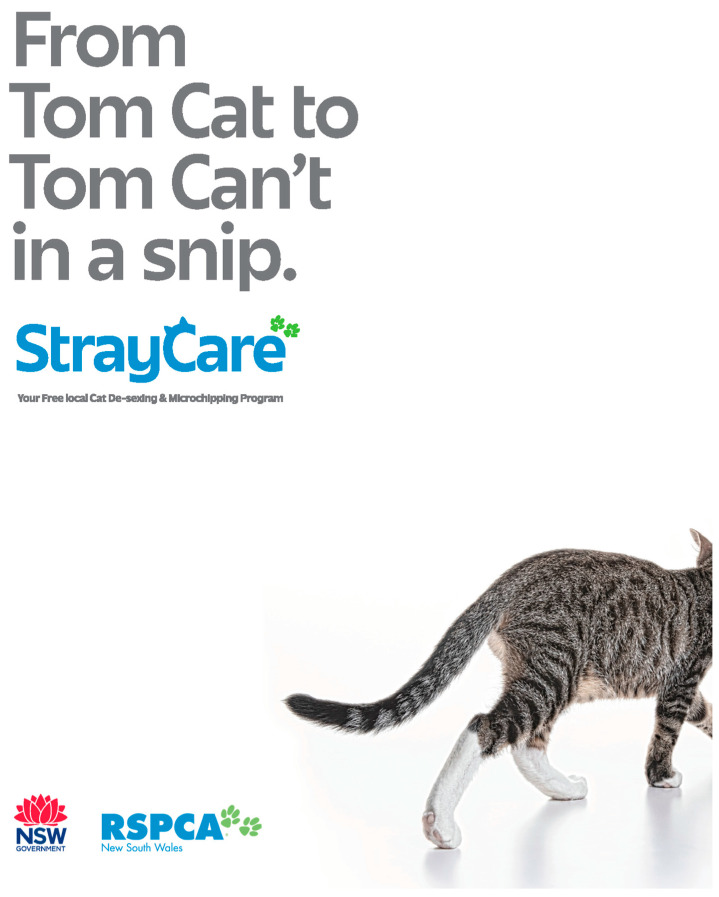
Example of a promotional poster for the StrayCare Metro targeted cat desexing program.

The StrayCare Metro program was run by RSPCA NSW and included free surgical sterilization (desexing) and microchipping for cats living within the participating LGA (Blue Mountains and Hornsby) or within selected target suburbs within a program LGA (Campbelltown and Parramatta). The program was promoted strategically to target caregivers of unowned free-roaming or ‘stray’ cats (semi-owners) and cat owners overwhelmed with multiple cats. Cats were eligible to be enrolled if they had a person able to provide consent for their desexing and who was designated as being responsible for their ongoing care. Consultation was undertaken with local council animal management officers, local animal welfare organizations, and cat rescue organizations to identify initial target suburbs, known multi-cat sites, and streets with populations of free-roaming cats without identified owners. Door-knocking and distribution of promotional flyers and posters in these areas identified additional semi-owners [34] and cat owners overwhelmed with multiple entire cats who were invited to participate in the program (Figure 2). All cats who were presented for desexing were scanned for a microchip, and where a microchip was detected, the registry details were checked to confirm they matched the details of the caregiver presenting the cat for surgery. Where microchip details did not match those of the caregiver, the cat was not desexed through the program unless consent was also received from the person on the microchip (their legal ‘owner’).

The program shared some similarities with programs described elsewhere as ‘Trap-Neuter-Return’ (TNR): the program specifically targeted caregivers of unowned cats, prioritized enrolling cats from multi-cat sites and unsocialized cats, and facilitated assistance with trapping where this was required because cats could not otherwise be caught and handled. Important differences between the StrayCare Metro approach and conventional TNR included: a broader scope including both owned and unowned cats; cats were only enrolled with the explicit consent of a designated caregiver who had a confirmed pre-existing relationship with the cat and who committed to their ongoing care and welfare; the program incorporated a human behavior-change approach, aiming to encourage caregivers of unowned cats to accept formal ‘ownership’ responsibility for the cats in their care. Where more than one caregiver was involved in the care of an unowned cat (which was common), engagement with the program commenced with a long-term planning discussion with the caregivers and other relevant stakeholders (e.g., the land managers and the local council) to decide who would take responsibility for the ongoing care of the cats and if any changes were required to improve their welfare and management.

**Figure 3:**
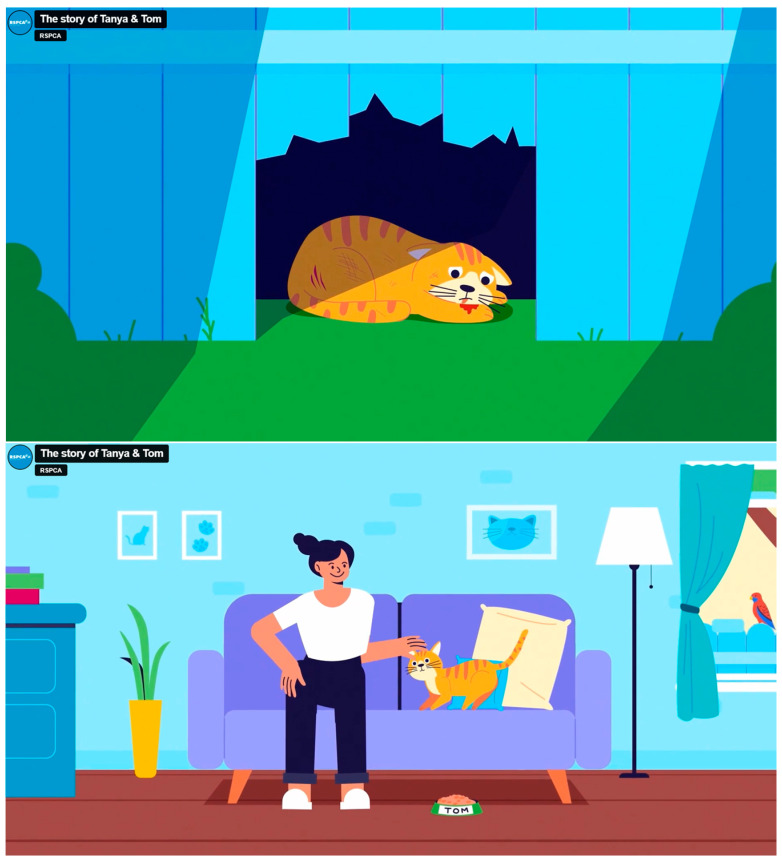
Stills from animated short film ‘Tanya and Tom’, which was used as part of the social marketing approach for the StrayCare Metro targeted cat desexing program to encourage everyone in the community to notice and act to help homeless cats in their neighborhoods.

The program was accompanied by a social marketing campaign through RSPCA NSW-owned Facebook, Instagram, LinkedIn, and TikTok channels, which encouraged the whole community to notice and act to help homeless cats in their neighborhoods (Figure 3). Key messages included:

- Every cat deserves a safe and permanent home with someone to care for them
- Found a cat? Make sure they have a home!
- Caring for stray cats takes the whole community working together.

Surgical sterilization of cats was performed by local private veterinary practices. Case management, assistance with transporting cats, and assistance with trapping unsocialized cats were facilitated where required through collaboration between RSPCA NSW, council animal management officers, cat rescue organizations, and/or local volunteers. The StrayCare Metro program was part of the RSPCA NSW project *Keeping Cats Safe at Home*, which used a human behavior-change approach to encourage cat caregivers to prevent their cats from roaming and increase uptake of desexing [46]. Funding for the program was provided by the NSW Government through its Environmental Trust through a 4-year grant.

### 2.3. Data Collection

The expected outcomes of the program on cat sub-populations and evaluation indicators were determined through discussion with stakeholders and experts (Table 1).

Data on council pound cat intake and euthanasia was collected from publicly available databases [47]. Participating councils provided records of cat-related nuisance complaints. Transect drives were used to estimate free-roaming cat population density.

### 2.4. Transect Drives

Budget and logistical constraints limited the number of transect drive sites to two LGAs. Campbelltown and Blue Mountains were selected to represent one heavily urbanized LGA with a large, relatively lower socio-economic human population and one less urbanized LGA with a more preserved natural environment and a higher socio-economic population. Methods are described in the study of Davey et al. [48]. In brief, transect drives followed an approximately 80 km route (excluding highways) through residential areas of each of the LGAs. Observations of free-roaming cats were recorded while driving the route at approximately 30 km/hr. The same route was repeated once daily, at the same time of day, on four separate days within a seven-day period in both Blue Mountains and Campbelltown. Using the same methods, transect drives were completed in 2021 and 2024.

### 2.5. Statistical Analysis

Associations between council pound cat intake and cat euthanasia before and after the program was implemented were modeled using negative binomial generalized linear models using the glm.nb() function from the MASS package in RStudio (Version 2025.05.1, Posit Software, PBC, Boston, MA, USA). The unit of analysis used in modeling was aggregated total values for each variable from the four program LGAs. ‘Year’ was treated as a continuous covariate, and missing years were handled via listwise deletion by the modeling function (no imputation). For the cat intake model, 11 yearly observations were included following listwise deletion of one year with missing data; 8 yearly observations were included for council euthanasia (no deletions indicated). Model adequacy was assessed using the estimated dispersion parameter (θ), residual deviance relative to degrees of freedom, AIC, and inspection of residual patterns; these diagnostics supported the use of the negative binomial specification over Poisson.

For transect drives, cat encounter rates (number of cats/km) were calculated by dividing the number of cats observed by the total sampling effort (km).

## 3. Results

### 3.1. Program Data

The StrayCare Metro program commenced in June 2022 and continued until December 2024. In total, 1225 cats were desexed (Table 2). Most cats were also permanently identified with the implantation of a microchip at the time of their surgical sterilization; 772 of 1072 cats (72%) were not already microchipped. Microchipping was offered free of charge and was recommended for all enrolled cats; however, because this was previously identified by the project team as a potential barrier to participation (Ma et al., 2023 [34]), microchipping was included only with the informed consent of the cat’s caregiver.

Most of the enrolled cats were passively acquired by their caregivers (86%); 63% were “a stray”, 13% were “gift or rehomed from a friend/relative/neighbor”, and 9% were the result of an unplanned litter. It was uncommon for enrolled cats to have previously been examined by a veterinarian; 86% were seeing a veterinarian for the first time when they were sterilized as part of the program. Cats under 6 months of age were the most represented cohort (38%), followed by cats aged 6–12 months (20%). However, the age of many participating cats was unknown (9%). According to their caregiver, one-third of participating adult female cats had previously had at least one litter of kittens. While most caregivers enrolled a single cat (66%), a small proportion of caregivers enrolled large numbers of cats. Five percent of caregivers enrolled more than five cats, including three individual caregivers who enrolled 27, 57, and 61 cats each. Almost half of the participating caregivers (46%) responded “yes” when asked, “do you have stray cats that visit where you live?”

### 3.2. Council Pound Cat Intake and Euthanasia

**Figure 4:**
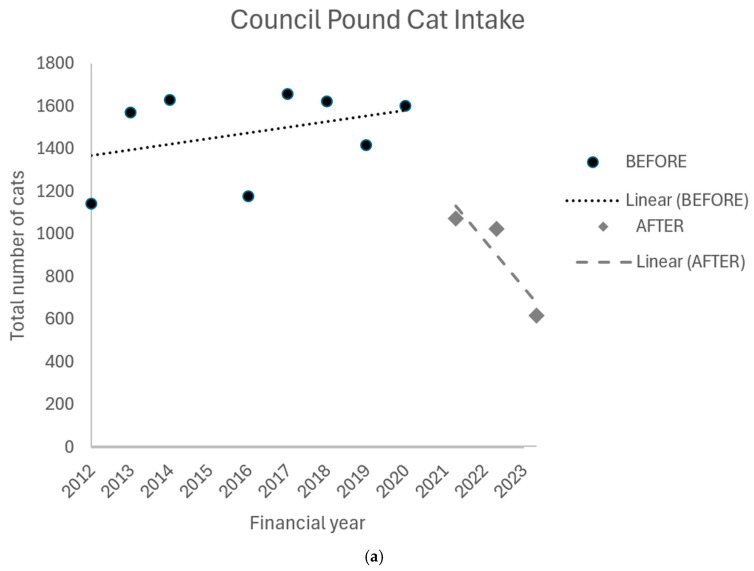

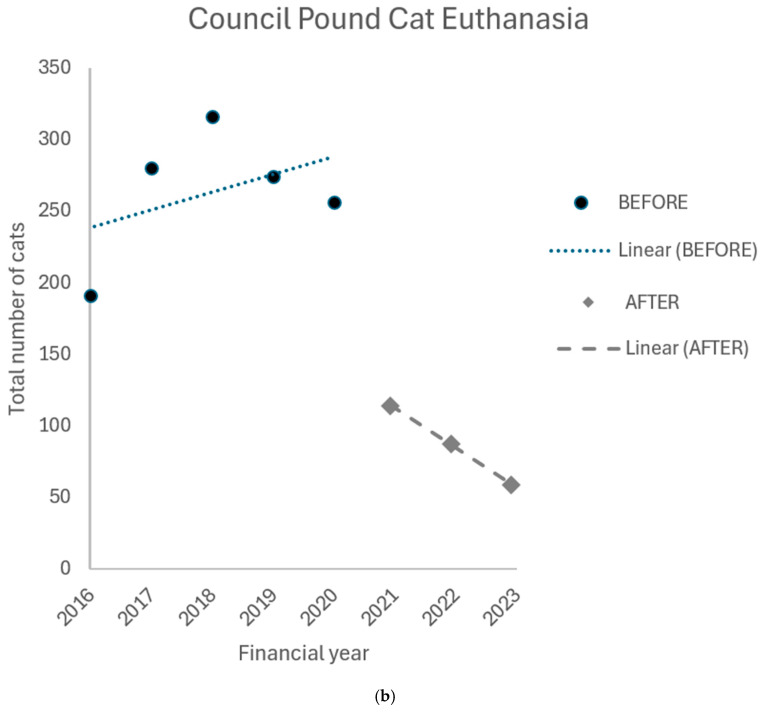
Total council pound cat intake (**a**) and cat euthanasia (**b**) for Blue Mountains City Council, Campbelltown City Council, Hornsby Shire Council, and City of Parramatta Council [34] before and after the StrayCare Metro targeted cat desexing program commenced in these areas in FY21-22.

Council pound cat intake varies considerably between local government areas in NSW. Campbelltown City Council consistently has amongst the highest council pound cat intake in the state, with an average annual cat intake of 995 cats between FY12-13 and FY20-21 (Table 3). Blue Mountains, City of Parramatta, and Hornsby respectively averaged 184, 242, and 82 cats annually during this period. Overall, the council pound cat intake and euthanasia for the four project council areas combined showed an upward trend between FY12-13 and FY20-21. The period after the implementation of the StrayCare Metro program saw a reversal of the upward trend in council pound cat intake (Figure 4a and Table 3) and euthanasia (Figure 4b and Table 4). Compared to the average annual cat intake for the four years before StrayCare activities commenced in FY21-22, the annual cat intake in FY23-24 decreased by 54% in Blue Mountains, 59% in Campbelltown, 73% in Parramatta, and 49% in Hornsby; a 61% overall reduction in annual council pound cat intake was observed across these four council areas. Similarly, compared to the average annual council pound cat euthanasia for the four years before StrayCare activities commenced in FY21-22, the number of cats euthanized in the council pounds decreased by 76% in Blue Mountains, 81% in Campbelltown, 98% in Parramatta, and 73% in Hornsby, or by 75% overall (Table 4). A negative binomial generalized linear model with a log link indicated that, after controlling for year, the intervention period was associated with a significant reduction in council pound cat intake (β = −0.546, SE = 0.173, z = −3.16, *p* = 0.002) and euthanasia (β = −0.951, SE = 0.273, z = −3.49, *p* < 0.001).

### 3.3. Council Cat-Related Nuisance Complaints

Cat-related nuisance complaint data were not collected routinely by the City of Parramatta Council until FY2020-21 and by Campbelltown City Council until FY2021-22. In the Campbelltown LGA, cat-related nuisance complaints decreased by 56% after one year of the StrayCare Metro program and by 64% after two years (Table 5). Nuisance complaints also decreased considerably in the Parramatta and Hornsby LGAs, where council animal management officers noticed improvements in their community:

*“Before the cat desexing program every second call we would get would be a cat job. Now after 12 months of desexing we haven’t had any cat jobs in about two months even though this would usually be the height of kitten season. Most of the cat jobs I have been getting lately have been owned and microchipped cats who were lost and I have been able to return to their homes. It has been a big change for the better.”*

—Council Animal Management Officer [50]

Conversely, the cat-related nuisance complaints in the Blue Mountains LGA increased in both the first and second years following the commencement of the StrayCare Metro program.

### 3.4. Transect Drives

Transect drives were undertaken between 2:30 and 5:30 pm on four days within a seven-day period over an 80 km route in both the Campbelltown and Blue Mountains LGAs in April 2021 and 2024. Encounter rates were considerably higher in Campbelltown (2.6 cats/km) compared to Blue Mountains (0.94 cats/km; Table 6). Encounter rates reduced substantially between 2021 and 2024 in both LGAs; by 51% in Blue Mountains and by 35% in Campbelltown.

## 4. Discussion

The StrayCare Metro program is an example of a One Welfare approach to cat population management, which emphasizes the interconnectedness of animal welfare, human well-being, and environmental health [51]. This study used an observational pre–post study design to evaluate changes associated with the StrayCare Metro targeted cat desexing program on indicators of cat welfare, cat population density and turnover, and cat-related nuisance complaints in four NSW local government areas (LGAs)s. As no control LGAs were included, all findings reflect comparisons within the participating LGAs over time, and direct causal effects cannot be confirmed. Over the two years following program implementation, the data show statistically significant reductions in council pound cat intake and euthanasia, along with reductions in cat-related nuisance complaints to council and estimated cat population density compared to pre-program measurements.

The holistic approach taken through the StrayCare Metro program aims to safeguard and improve cat welfare, protect wildlife and the environment, and address the needs of humans and, in doing so, ensure that interventions are sustainable and beneficial for all stakeholders [38,51,52]. Targeted desexing programs can reduce cat overpopulation, resulting in fewer cats subject to potential hazards and welfare compromises that free-roaming cats can face (e.g., disease, malnutrition, and injuries) and improve the likelihood that cats can stay with their human caregivers [1,22,53,54,55]. This can not only improve the overall welfare of the cat population but can also reduce the burden on council pounds, animal shelters, and rescue organizations [22]. Protection of native wildlife from the potential negative impacts of cats and reducing the spread of cat-related zoonotic diseases are additional strong motivators for effectively managing cat populations [10,33]. The One Welfare approach taken by the StrayCare Metro program can support people to keep the cats they are already caring for, foster a sense of community responsibility and engagement, and promote collaboration and shared responsibility between local councils, animal welfare organizations, and community members while operating within the existing NSW regulatory framework [22,43].

Our findings support a role for well-resourced, holistic, and collaborative cat desexing programs to contribute to achieving desired outcomes for cat management, with potential community and ecological benefits. Collaboration with council animal management workers, animal welfare organizations, and cat rescue organizations as part of the StrayCare Metro program are hypothesized to have contributed to rapid reductions in council pound cat intake (49–73% reduction over 2 years) and even more dramatic reductions in cat euthanasia (73–98% reduction) in all project areas, and a reduction in cat-related complaints in all but one project area (47–64%). The population reductions seen might have been partially attributable to the removal of cats through rehoming (especially of kittens and highly socialized healthy adults) and euthanasia (where appropriate and with informed caregiver consent), in addition to desexing both owned and unowned cats directly through the StrayCare Metro program and natural attrition. Removal of cats through rehoming and euthanasia was not formally a component of the StrayCare Metro program; hence, it was not described in this study but was often facilitated by RSPCA NSW through local stakeholders as a result of engaging with cat caregivers through the StrayCare Metro program. The combination of desexing with removal of cats has been shown to be more effective than desexing of cats alone [43,56,57]. The decrease in cat intake to council pounds, cat euthanasia, and cat-related complaints observed in this study is consistent with other similar programs that took an assistive and One Welfare-focused approach to cat management [22,31]. For example, the pilot Rosewood Community Cat Program in Queensland, Australia, in which cat intake to animal shelters from the targeted suburbs decreased by 60% and numbers of cats euthanized by 85% over three years [30]. Another example in Victoria, Australia, saw a reduction in cat impoundments of 66% and euthanasia of 82%, and a decrease in cat-related calls to the local council over 8 years [22]. In all three programs, the dual focus on desexing both owned and unowned cats theoretically allows breeding to be directly addressed within the unowned population while simultaneously reducing recruitment into this population from the owned cat population, which can occur via straying and/or abandonment of entire owned cats and unwanted litters of kittens. This is consistent with modeling evidence demonstrating the importance of desexing both owned and unowned cats in broader cat population control [41].

Certain elements of the StrayCare Metro program design are considered to have contributed to the outcomes observed. The program relied heavily on the expertise, relationships, and local knowledge of council animal management officers, cat rescue groups, and community volunteers. This decentralized approach was supported by a case management model, which prioritized identifying and following up on multi-cat sites, whether or not these cats were considered ‘owned’ by their caregiver. Following evidence from previous studies [22], the StrayCare Metro program did not limit the number of cats that could be enrolled per caregiver. Instead, priority was given to achieving high sterilization rates at each multi-cat site as quickly as possible, noting that the previous literature has modeled that over 70% of breeding animals need to be sterilized to prevent population increases [18,58]. Multi-cat sites were identified through local stakeholder engagement and further targeted with letterbox drops aiming to ‘mop up’ cats that may have dispersed from the multi-cat site into surrounding areas. Desexing was conducted through local veterinary practices to increase service accessibility, and assistance was facilitated to transport cats where required, recognizing that transport can be a significant barrier [34,59]. This design also aligns with insights from analyses of community cat management, demonstrating how strategic, context-specific planning and a One Welfare approach can enhance intervention outcomes [41,43,60,61]. The incorporation of a human behavior-change framework, embedded within the larger *Keeping Cats Safe at Home* social marketing campaign, encouraged ’responsible‘ cat guardianship, while mobilizing the wider community to notice and actively manage unowned cats without relying solely on council or RSPCA NSW intervention. Increasing the uptake of so-called ‘responsible’ cat guardianship behaviors such as desexing and containment can help to reduce the birth of unwanted litters of kittens and straying and abandonment of owned cats, thereby reducing the contribution of the owned cat population to the unowned cat population [41].

Identifying and case-managing multi-cat sites appears to be a critical element of effective cat population management. Caregivers with large numbers of cats—whether overwhelmed owners or individuals caring for unowned cats—often face multiple, complex barriers to accessing veterinary services and desexing the cats they care for. These multi-cat situations intersect with understandings of the various forms of animal hoarding [62]. In our experience, multi-cat sites—while often relatively few in number—disproportionately contribute to cat management challenges within a given area, such as shelter cat intake and nuisance concerns. Caregivers of multi-cat sites often have long-standing antagonistic relationships with their local council and animal welfare organizations [38,63]. Previous instances where cats have been seized and killed can erode trust while failing to address the root issue [64]. Instead, a collaborative case management approach appears to be more effective, in which council and animal welfare organization representatives work alongside these complex situations to desex all cats at the site, reduce numbers to a sustainable level if appropriate, and stabilize the population. Targeted, sustained intervention at these sites not only addresses immediate welfare concerns for both the cats and humans involved but also prevents ongoing population growth and dispersal into the surrounding community.

Accessible surrender options to an animal welfare or cat rehoming organization represent a potentially impactful component of unowned cat management programs, providing a practical means for humanely removing cats from the free-roaming population and hence achieving quicker reductions in population density and associated problems. This has been supported by cat population modeling studies [18,41]. Such options can facilitate more rapid improvements in both human and cat welfare by alleviating overcrowding at overpopulated sites and reducing the caregiving burden on individuals who are overwhelmed [34]. In addition, when surrender options are accessible, shelter cat intake data better reflect cat populations and can be used to plan and target support programs to areas of greatest need. To be effective and ethical, this approach must be collaborative, working closely with caregivers to ensure that any actions are undertaken with fully informed caregiver consent. This is particularly important when considering the removal, euthanasia (ending the cat’s life to relieve unavoidable suffering), or humane killing of cats (where cats are killed rapidly and without pain or distress for reasons other than to relieve suffering). Multiple studies demonstrate the strong attachments between caregivers and unowned cats [35,38,64,65]. Our anecdotal experience at RSPCA NSW suggests that accessible surrender options are especially important in situations where a person who has been caring for a group of cats—particularly unsocialized individuals—becomes unable to continue due to relocation, declining health, or death. Without an accessible surrender pathway, these cats are likely to suffer poor welfare and to disperse to seek food and shelter, potentially creating new problem sites. Where possible, in situ management of unsocialized cats who are sterilized and receive ongoing care might lead to improved cat welfare outcomes [66]. However, where no such committed caregiver exists or where a committed caregiver can no longer care for the cat/s, surrender should remain an available and supported pathway.

Many caregivers declined microchipping for enrolled cats, even when this was offered free of charge (28% of non-microchipped enrolled cats). This refusal is noteworthy as the *Companion Animals Act 1998* (NSW) mandates that all cats in NSW be permanently identified and registered by 12 weeks of age or on transfer of ownership. The high rate of microchip refusal in our study likely reflects economic and ownership-perception barriers. For the caregivers of unowned cats, the compulsory one-off registration fee (AUD$70 in 2025) presents a tangible cost barrier to formalizing ownership, especially for caregivers of multiple cats. This legal requirement to register cats was an important deterrent to microchipping some cats through the program, even though microchipping was offered free of charge. This is supported by Australian research that demonstrates that financial barriers to sterilization, microchipping, and registration are more prevalent in disadvantaged communities and contribute to the persistence of unowned cat populations [52]. Given that registration requirements were introduced to support animal welfare, owner accountability, and reunification of lost animals, the reluctance of semi-owners to engage may undermine those regulatory goals.

The changes observed in cat-related nuisance complaints varied between councils, with some experiencing substantial reductions, while complaints increased in the Blue Mountains. In councils where complaints declined, possible contributing factors associated with the StrayCare program included a reduction in roaming cats—particularly unowned kittens—and the existence of a clear pathway for residents to address concerns. This may have reassured communities that the problem was being actively managed. In contrast, increases in complaints in Blue Mountains may have been influenced by heightened public awareness generated by the program’s educational and containment messaging through the *Keeping Cats Safe at Home* initiative. In the Blue Mountains, differences in local communication strategies, including an emphasis on the impacts of roaming cats on wildlife, and a relatively smaller unowned cat problem, but a larger proportion of owned roaming cats, may have heightened concern and reporting rates. Nonetheless, even if more complaints are received, if there is an effective and straightforward way for councils to respond (for example, by referring complainants to programs like StrayCare Metro), the overall outcome may be positive, and complaints should reduce over time. For this reason, monitoring satisfaction with complaint responses might also be helpful to monitor. The absence of differentiation in council data between complaints about owned versus unowned cats limits interpretation and underscores the need for more detailed and standardized nuisance complaint recording by councils. Ideally, cat-related nuisance reporting would distinguish between complaints about unowned (or ‘stray’) cats and those concerning owned cats, to better evaluate program outcomes and guide targeted interventions. The variation in changes across different LGAs suggests that community-specific factors must be considered when designing and implementing similar programs, including tailored communication and engagement strategies in different communities.

This study demonstrated a reduction in cat population density estimates of 35–50% between 2021 and 2024 in the two areas in which transect drives were used to monitor cat populations. Transect drives and other street-based survey methods adapt established ecological sampling techniques and are recommended by organizations such as the International Coalition for Companion Animal Management (ICAM) as feasible and repeatable approaches for estimating absolute dog or cat population sizes and/or for monitoring relative population metrics (e.g., encounter rates and counts) [67,68]. To our knowledge, this is the first report of using transect drives as part of the monitoring and evaluation for an intervention on domestic cat populations in Australia. Although these methods can only estimate cat populations, if used appropriately and consistently, they can provide a more direct assessment of trends in cat density associated with management efforts. This information can then be correlated with other measures of interest that are directly related to desired outcomes of cat management such as benefits to the community (e.g., reduced cat intake to shelters and council pounds, and cat-related complaints) and biodiversity (e.g., recovery or maintenance of vulnerable native wildlife populations). Although the measures of ‘success’ for cat management programs vary widely [43], objective monitoring of agreed outcome measures linked to the specific aims of cat management is key to an appropriate adaptive management approach that focuses on optimizing the use of limited resources to achieve the desired outcomes humanely and effectively. The importance of objectively monitoring cat populations and linking this with native wildlife outcomes in Australia has also been identified as vital for guiding government investment [69].

### Limitations and Future Directions

Interpretation of the program’s outcomes must be considered in light of several limitations and potential confounders. The study was conducted in only four local government areas (LGAs) within the Greater Sydney region and did not include control LGAs, which may limit the generalizability of the findings to other regions with different socio-economic, environmental, and demographic characteristics. Although aggregated analyses in this study showed overall reductions in pound cat intake and euthanasia across the participating LGAs, the magnitude and direction of these changes were not uniform. For example, fluctuations in euthanasia numbers in the Blue Mountains between 2022 and 2023 and between 2023 and 2024 highlight that local factors continued to influence year-to-year outcomes, underscoring the need to interpret program impacts within the context of inter-LGA heterogeneity rather than relying solely on pooled totals. Additionally, the selection of transect drive sites was limited to two LGAs due to budget and logistical constraints, which may not fully represent the variability in cat population densities across all participating LGAs. Future studies could explore the effectiveness of targeted desexing programs in different socio-economic, environmental, and demographic contexts beyond the Greater Sydney region. This would help determine the generalizability of the findings and identify any region-specific factors that may influence program outcomes, including the motivations and barriers faced by cat caregivers in different communities [9].

Reduced council pound cat intakes observed might in part reflect changes in intake protocols rather than genuine shifts in cat populations. Additionally, council behaviors—such as referring cats to the desexing program instead of accepting them as surrenders—likely contributed to observed declines in intake and euthanasia figures. These factors may have affected the observed reductions in cat intake and euthanasia, making it difficult to attribute these changes solely to the StrayCare Metro program. It is important to assess the impact of policy and legislative changes on cat management practices and outcomes, as this could provide insights into the role of governance in supporting effective interventions.

Other unmeasured factors, including climatic variation, local government approaches to cat management, and levels of community engagement, could also have influenced cat numbers, cat intake and euthanasia in pounds and shelters, and cat-related calls to councils. Societal trends (e.g., towards greater uptake of cat containment and sterilization), administrative changes (e.g., within local councils and animal shelters), and other factors that varied over the same time period as the program might also have contributed to observed reductions. Future work should consider intervention modeling that accounts for these factors to help to optimize program design and targeting in diverse settings. Conducting long-term studies to monitor the sustainability program impacts over several years would provide valuable insights into the long-term effectiveness of targeted desexing interventions. This could include tracking changes in cat population dynamics, welfare indicators, and community attitudes over time.

## 5. Conclusions

This study observed reductions in council pound cat intake, euthanasia, cat-related nuisance complaints to council, and reductions in cat encounter rates after implementation of the StrayCare Metro free cat desexing program, supporting its use in pursuing cat management outcomes. The pre–post observational design of this study limits attribution of these observed changes directly to the implementation of the program. However, the holistic and collaborative approach of the program, its focus on balancing benefits for all stakeholders, including cats and their caregivers, and the incorporation of human behavior-change and social marketing elements might have contributed to its efficacy. To sustain gains and mitigate the risk of cat population rebound in subsequent years, ongoing investment in desexing access, community engagement, and rehoming capacity—rather than time-limited funding—will be essential.

## Figures and Tables

**Table 1:** StrayCare Metro targeted cat desexing program evaluation outcomes and indicators.

Outcome	Indicator	Source
Improve cat welfare	Council pound cat intake	Office of Local Government
Reduce cat overpopulation	Free-roaming cat population density	Transect drives
Reduce cat population turnover	Shelter cat intake	Office of Local Government
	Cat euthanasia	Office of Local Government
Reduce cat-related nuisance	Cat-related nuisance complaints	Council

**Table 2:** Features and program outputs for four local government areas (LGAs) participating in the StrayCare Metro targeted cat desexing program between June 2022 and December 2024.

LGA	Land Area (ha), % Residential ^1^	Estimated Resident Population ^1^	Occupied Private Dwellings ^1^	Population Density (People per 1000 ha)	Cats Sterilized	Intervention Intensity–Population ^2^	Intervention Intensity–Private Dwellings ^3^	Intervention Intensity–Land Area ^4^
Blue Mountains	143,114.4 (12%)	78,121	30,528	546	169	2.2	5.5	9.8
Campbelltown	31,141.2 (20%)	176,519	57,368	5668	500	2.8	8.7	80
Hornsby	45,503.7 (16%)	151,811	51,629	3336	180	1.1	3.4	25
Parramatta	8383 (58%)	256,729	92,109	30,625	382	1.5	4.1	79

^1^ 2021 Australian Census of Population and Housing [49], ^2^ number of cats sterilized per 1000 estimated resident population, ^3^ number of cats sterilized per 1000 occupied private dwellings, and ^4^ number of cats sterilized per 1000 ha of land classified as residential.

**Table 3:** Total cat intake to the local council pound for StrayCare Metro local government areas (LGAs).

LGA	2012–2013	2013–2014	2014–2015	2015–2016	2016–2017	2017–2018	2018–2019	2019–2020	2020–2021	2021–2022	2022–2023	2023–2024
Blue Mountains	214	188	172	*	196	171	148	183	200	98	97	80
Campbelltown	901	1065	1115	*	686	1112	1052	895	1135	774	706	433
Hornsby	*	294	322	*	218	288	289	243	150	47	111	45
Parramatta	27	23	21	*	80	86	135	96	118	151	109	56
Total	1142	1570	1630	*	1180	1657	1624	1417	1603	1070	1023	614

* data missing.

**Table 4:** Number of cats euthanized at the local council pound for StrayCare Metro local government areas (LGAs).

LGA	2016–2017	2017–2018	2018–2019	2019–2020	2020–2021	2021–2022	2022–2023	2023–2024
Blue Mountains	43	37	18	29	34	15	7	35
Campbelltown	35	42	63	51	38	28	37	9
Hornsby	63	140	145	138	99	10	11	3
Parramatta	50	61	90	56	85	77	45	20
Total	191	280	316	274	256	130	100	67

**Table 5:** Cat-related nuisance complaints to the local council each financial year and the change in nuisance complaints following the commencement of the StrayCare Metro targeted cat desexing program.

Program	2017–2018	2018–2019	2019–2020	2020–2021	2021–2022	2022–2023	2023–2024	% Change ^1^ Year 1	% Change ^1^ Year 2
Blue Mountains	18	26	25	32	32	34	45	+35	+78
Campbelltown	-	-	-	-	501	222	180	−56	−64
Hornsby	133	228	186	255	241	142	107	−29	−47
Parramatta	-	-	-	43	59	26	21	−49	−59

^1^ Compared to the annual average for the four years FY2017-18 to FY2020-21 for Blue Mountains and Hornsby, compared to FY2021-22 for Campbelltown, and compared to the average of FY2020-21 and FY2021-22 for Parramatta.

**Table 6:** Cat encounter rate estimates before and after implementation of the StrayCare Metro targeted cat desexing program in Blue Mountains and Campbelltown local government areas calculated using line transect sampling.

LGA	Effort (km)	Cats Observed	Encounter Rate (Cats/km)
2021			
Blue Mountains	80	75	0.9375
Campbelltown	80	205	2.5625
2024			
Blue Mountains	80	37	0.4625
Campbelltown	80	133	1.6625

LGA = Local Government Area.

## Data Availability

All data used in analyses in this study is included within the manuscript.
